# Sleep onset problems and problems maintaining sleep among adults in Germany. Results from the Panel ‘Health in Germany’ 2024

**DOI:** 10.25646/14234

**Published:** 2026-06-03

**Authors:** Ann-Kristin Beyer, Dinara Yessimova, Robert Schlack, Hannelore Neuhauser, Julia Nübel

**Affiliations:** Robert Koch Institute, Department of Epidemiology and Health Monitoring, Berlin, Germany

**Keywords:** Female, Adults, Prevalence, Sleep-wake disorders, Sleep onset problems, Problems maintaining sleep, Surveys and questionnaires, Panel, Germany

## Abstract

**Background:**

Sleep problems can have negative consequences for quality of life, functioning and health. This fact sheet investigates the prevalence of sleep onset problems and problems maintaining sleep among adults in Germany in 2024.

**Methods:**

In the Robert Koch Institute’s panel ‘Health in Germany’, n = 27,038 participants (51.1 % female) were surveyed on the frequency of sleep onset problems and problems maintaining sleep over the past four weeks. The analyses were stratified by gender, age and education group.

**Results:**

Overall, 16.3 % of participants reported sleep onset problems and 31.7 % reported problems maintaining sleep. Differences were found across gender, age and education group, with higher prevalence among women and individuals in the low education group.

**Conclusions:**

Around one in three adults in Germany reports sleep onset problems or problems maintaining sleep. The results highlight the high public health relevance of problems with falling asleep and staying asleep.

## 1. Introduction

Many people are familiar with sleep problems. They often manifest as problems falling asleep (sleep onset problems) or waking up during the night with prolonged periods of wakefulness and difficulty getting back to sleep (problems maintaining sleep) [1–4]. Depending on the severity and duration, disturbed sleep can be accompanied by symptoms such as tiredness, and problems with attention, concentration or memory. If sleep problems occur regularly over a prolonged period and impair performance or quality of life, a sleep disorder (insomnia) may be present [5]. Insomnia can have various organic and/or non-organic causes and is a risk factor for physical illnesses (particularly cardiovascular diseases) and mental disorders [6, 7]. Furthermore, it is associated with increased healthcare utilisation and higher direct costs (e.g. for diagnosis and treatment), as well as indirect costs (e.g. due to reduced productivity at work or short- and long-term work incapacity) [8, 9].

Sleep onset problems and problems maintaining sleep are widespread; however, there is no current population-based data available for Germany. The aim of this fact sheet is to examine the prevalence of self-reported sleep onset problems and problems maintaining sleep among adults in Germany, based on data from 2024.

**Key messages**

► In 2024, around 20 % of women reported sleep onset problems and 36 % reported problems maintaining sleep.
► Almost 13 % of men were affected by sleep onset problems in 2024, and 27 % reported problems maintaining sleep.
► Both sleep onset problems and problems maintaining sleep were more prevalent among individuals in the low education group than among those in the high education group.

## 2. Methods

### 2.1 Study design and sample

Data from the Robert Koch Institute (RKI) panel ‘Health in Germany’ were used for the study. For the 2024 annual wave, the RKI Panel comprised 46,863 registered participants aged 18 and over (data set version 5). In 2024, they were invited to participate in health surveys at three points in time (sub-waves) at intervals of around two months. In total, there were four different questionnaires on different topics [10]. Participation was possible online and in writing by post. Data collection took place from May 2024 to January 2025. The response rate was between 75 % and 81 % in the individual sub-waves according to the standards of the American Association for Public Opinion Research (AAPOR [11]). A detailed description of the methodology can be found in Lemcke et al. [12].

### 2.2 Indicators

Sleep onset problems and problems maintaining sleep were assessed using two questions. Participants were asked how often they had problems falling asleep and staying asleep in the last four weeks. The response options ‘not at all’, ‘less than once a week’, ‘once or twice a week’, and ‘three times a week or more’ were dichotomised, with an occurrence of three times a week or more indicating sleep onset problems or problems maintaining sleep according to the ICD-10 [13, 14].

Gender, age and education group were used as stratification variables. The analyses by gender include people who identify as female or male. Gender-diverse people (n = 64) were not shown separately in the analyses by gender due to the small number of cases. The age of participants at the time of the survey was divided into five age groups: 18 to 29, 30 to 44, 45 to 64, 65 to 79, and 80 years and over. The education group was categorised as low, medium and high based on educational data in accordance with the CASMIN classification (Comparative Analysis of Social Mobility in Industrial Nations [15]).

### 2.3 Statistical methods

The prevalence of sleep onset problems and problems maintaining sleep is displayed as percentage with 95 % confidence intervals. The results are reported for the total population as well as separately for women and men and by age and education groups.

The analyses were conducted using R version 4.3.0 and RStudio version 2025.05.1. The difference in prevalence between the groups was considered statistically significant if the 95 % confidence intervals did not overlap. In order to correct for distortions due to selective participation and deviations of the sample from the population structure, the analyses were conducted using weighting. A detailed description of the calculation of the multi-stage sample weight can be found in Damerow et al. [16].

**RKI Panel ‘Health in Germany’ 2024**

**Data holder:** Robert Koch Institute

**Objectives:** To provide comprehensive data on the health status, health-related behaviour and health care of the population in Germany, with the future possibility of longitudinal comparisons and analysis of trends over time

**Study design:** Panel study with a mixed-mode approach (online and written-postal participation)

**Population:** German-speaking population aged 18 and over in private households with main residence in Germany

**Sample:** Probabilistic/representative sample of the ‘Health in Germany’ panel infrastructure

**Participants in the 2024 annual wave:** A total of 41,376 of the persons registered in the panel took part in at least one of the three sub-waves in 2024.

Questionnaire A: 14,759 women, 12,374 men,

66 persons with other gender identities

Questionnaire B: 14,828 women, 12,258 men,

61 persons with other gender identities

Questionnaire C: 14,709 women, 12,329 men,

64 persons with other gender identities

Questionnaire D: 14,872 women, 12,368 men,

66 persons with other gender identities

**Data collection:**

1st sub-wave: 28.05.2024 – 05.08.2024

2nd sub-wave: 12.08.2024 – 14.10.2024

3rd sub-wave: 28.10.2024 – 06.01.2025

More information at www.rki.de/panel-en

## 3. Results

Sleep onset problems and problems maintaining sleep were assessed in the RKI Panel in Questionnaire C, which was completed by 27,038 participants (51.1 % female) aged between 18 and 99 years. On average, the participants were 51.4 years old, 33.3 % had a low level of education and 20.3 % a high level of education.

Sleep onset problems were reported by 16.3 % of participants, women were affected more frequently than men (19.7 % vs. 12.8 %, Table 1). The frequencies were comparable across all age groups, except for women over 80, who were affected more frequently (26.5 %) than women in all other age groups, and men aged 65 – 79 (9.5 %), who reported sleep onset problems less frequently than younger men. An education gradient was also observed: women and men in the low education group were affected most frequently (women: 25.4 %, men: 16.6 %), whilst participants in the high education group were affected least frequently (women: 10.9 %, men: 7.3 %).

Problems maintaining sleep were reported by 31.7 % of participants. Women were affected more frequently than men (36.0 % vs. 27.1 %, Table 1). With regard to age groups, it was found that women in younger adulthood reported problems maintaining sleep less frequently than those in middle and older adulthood. Women aged 45 to 64 and those over 80 were most frequently affected by problems maintaining sleep. Among men, there was an increase in problems maintaining sleep with increasing age in the younger age groups (18 – 29-year-olds: 15.1 %, 30 – 44-year-olds: 22.7 %); from the age of 45 onwards, approximately one in three men reported problems maintaining sleep. Women in the low or medium education group (39.4 % and 36.2 % respectively) reported problems maintaining sleep more frequently than women in the high education group (29.2 %). Men in the low education group were affected more frequently (32.5 %) than men in the medium or high education group (24.8 % and 23.6 % respectively).

Overall, 35.3 % of participants reported sleep onset problems and/or problems maintaining sleep (women: 40.0 %, men: 30.4 %). Both sleep onset problems and problems maintaining sleep were reported by 12.6 % of participants (women: 15.4 %, men: 9.7 %).

## 4. Discussion

Data from the RKI Panel ‘Health in Germany’ 2024 show that around one in six adults report sleep onset problems and almost one in three report problems maintaining sleep. Around one in eight adults is affected by both sleep onset problems and problems maintaining sleep. In general, the comparability of different surveys on symptoms depends on the wording or definition of the symptoms – for example, regarding duration, frequency and timeframe – but also on the investigated sample. An international review of studies from 1997 to 2015 confirms that problems maintaining sleep are significantly more common than sleep onset problems, but reports lower prevalence, particularly for problems maintaining sleep [1]. Lower frequencies are also found in previous national data: a survey conducted on behalf of the health insurance ‘Techniker Krankenkasse’ in 2017 showed a prevalence of 13 % for self-reported sleep onset problems and 24 % for problems maintaining sleep [2]. A survey conducted on behalf of the health insurance ‘BARMER’ in 2018 showed a prevalence of 21 % for self-reported sleep onset problems and problems maintaining sleep [3]. In the RKI study ‘German Health Interview and Examination Survey for Adults’ (DEGS 1), using data from 2008 – 2011, the overall prevalence of sleep onset problems and/or problems maintaining sleep among 18- to 79-year-olds was 5 percentage points lower than the prevalence in this analysis (30.3 % vs. 35.3 %), based on an identical questionnaire. The prevalence of sleep onset problems was also significantly lower in 2008 – 2011 than the current estimate (11.1 % vs. 16.3 %); this also applies to problems maintaining sleep (23.0 % vs. 31.7 %) [4]. The differences primarily affect women and men in the younger and middle age groups. An increase in sleep-related complaints over time would be conceivable in the context of increased media use, particularly of smartphones, or multiple societal crises, for example as a consequence of the COVID-19 pandemic, climate change or looming military conflicts. Accordingly, there was also a significant increase in depressive symptoms, which can be associated with sleep problems, among adults in Germany between 2021 and 2023 [17]. Similarly, data from the health insurance ‘Kaufmännische Krankenkasse’ showed a significant increase in medically diagnosed nonorganic sleep disorders since 2014, with the sharpest increase occurring in 2024 compared to the previous year [18].

Our findings reveal differences according to gender, age and education group, as reported in other studies [19]. In middle age, occupational and psychosocial factors, such as shift work, stress and worries, can have a negative impact on sleep. Furthermore, as people age, the likelihood of chronic conditions, medication use or pain increases, all of which can disrupt sleep. Changes in daily routines due to the transition into retirement can also influence sleep patterns [20, 21]. In women, sleep problems may also be caused by hormonal changes (e.g. during pregnancy or the menopause). Furthermore, women have a higher risk of mental disorders, such as depression or anxiety disorders, which are associated with sleep disturbances and may also include sleep problems as part of their symptoms [22]. A lower level of education is also often associated with increased stress, a higher likelihood of physical or mental health conditions, and limited access to healthcare, which in turn increases the risk of sleep problems [23].

Given the considerable health, social and economic importance of good sleep, the findings highlight the high public health relevance and the importance of preventive measures for sleep onset problems and problems maintaining sleep. These include individual measures such as promoting good sleep hygiene (e.g. keeping a regular sleep schedule, avoiding alcohol, nicotine and caffeine, and refraining from using media or smartphones before bedtime). Furthermore, structural measures can also help prevent sleep problems, such as those addressing environmental factors (e.g. noise and heat protection) and work-related aspects (e.g. reducing shift work and chronic work-related stress) – as well as complex stress factors, such as social inequality [24, 25].

If sleep onset problems and problems maintaining sleep persist over a longer period and significantly impair performance in everyday life and quality of life, those affected should seek medical advice to determine whether a medically defined sleep disorder (insomnia) is present and what treatment options are available – also with regard to potentially underlying physical or mental health conditions [5]. Further studies are needed to assess the long-term trends in sleep onset problems and problems maintaining sleep in Germany.

## Figures and Tables

**Table 1:** Prevalence of sleep onset problems (n = 14,434 women; n = 12,145 men) and problems maintaining sleep (n = 14,502 women; n = 12,179 men) by gender, age and education group. Source: RKI Panel 2024

Sleep onset problems	%	(95 %-CI)		%	(95 %-CI)
Total (women and men)	16.3	(15.7 – 17.0)			
**Women (total)**	**19.7**	**(18.8 – 20.7)**	**Men (total)**	**12.8**	**(11.9 – 13.7)**
**Age group**			**Age group**		
18 – 29 years	19.8	(17.3 – 22.5)	18 – 29 years	13.9	(11.6 – 16.4)
30 – 44 years	17.0	(15.2 – 19.0)	30 – 44 years	13.1	(11.2 – 15.2)
45 – 64 years	20.0	(18.5 – 21.6)	45 – 64 years	13.6	(12.2 – 15.2)
65 – 79 years	19.0	(17.3 – 20.9)	65 – 79 years	9.5	(8.2 – 10.9)
80 + years	26.5	(22.9 – 30.4)	80 + years	13.1	(10.4 – 16.3)
**Education group**			**Education group**		
Low	25.4	(23.2 – 27.7)	Low	16.6	(14.7 – 18.7)
Medium	19.3	(18.2 – 20.4)	Medium	12.7	(11.5 – 14.0)
High	10.9	(9.8 – 12.1)	High	7.3	(6.3 – 8.3)
**Problems maintaining sleep**	**%**	**(95 %-CI)**		**%**	**(95 %-CI)**
Total (women and men)	31.7	(30.9 – 32.4)			
**Women (total)**	**36.0**	**(34.9 – 37.1)**	**Men (total)**	**27.1**	**(26.0 – 28.2)**
**Age group**			**Age group**		
18 – 29 years	25.5	(22.9 – 28.4)	18 – 29 years	15.1	(12.7 – 17.9)
30 – 44 years	32.4	(30.2 – 34.7)	30 – 44 years	22.7	(20.5 – 25.0)
45 – 64 years	40.6	(38.8 – 42.5)	45 – 64 years	33.0	(31.1 – 35.0)
65 – 79 years	36.2	(34.1 – 38.5)	65 – 79 years	29.8	(27.8 – 31.9)
80 + years	43.6	(39.6 – 47.8)	80 + years	35.8	(32.1 – 39.7)
**Education group**			**Education group**		
Low	39.4	(37.0 – 41.9)	Low	32.5	(30.1 – 34.9)
Medium	36.2	(34.9 – 37.6)	Medium	24.8	(23.4 – 26.3)
High	29.2	(27.6 – 30.9)	High	23.6	(22.2 – 25.2)

CI = confidence interval
